# A computational study on off-center rotational dynamics of human semicircular canals with implications for real and virtual worlds

**DOI:** 10.1016/j.heliyon.2024.e41035

**Published:** 2024-12-06

**Authors:** Sion Cha, Wooksung Kim

**Affiliations:** Department of Electrical Engineering, Pohang University of Science and Technology (POSTECH), 77 Cheongam-Ro, Nam-Gu, Pohang, Gyeongbuk, 37673, Republic of Korea

**Keywords:** Rotational perception, Sensory conflict, Spatial orientation, Eccentric rotation, Vestibular system, Finite element method, Fluid-structure interaction

## Abstract

This study investigates human semicircular canal (SCC) dynamics under off-center rotational conditions. Previous research has modeled human rotational perception and the dynamic response of the SCCs by assuming a centered rotation state, where the rotation axis aligns with the SCC's center. However, this assumption is not representative of most real-life rotational situations. Understanding the effect of the offset distance between the rotation axis and the centers of the SCCs is essential, yet many studies still rely on bandpass filter models that do not account for this factor. Experimental studies are also limited, and mock-up models have difficulty accurately depicting these dynamics due to the cupula's low Young's modulus. Therefore, this study models endolymph and cupula within the SCCs using the finite element method (FEM) and a two-way fluid-structure interaction (FSI) approach. The results compare cupula displacement across different rotational conditions: step velocity motion (SVM), step acceleration motion (SAM), and sinusoidal motion. Notably, as the offset distance increases, the gain factor increases while the long time constant decreases. This finding highlights the limitations of existing centered rotation-based bandpass filter models. Based on these findings, we propose a modified transfer function that accounts for offset distance, offering a more generalizable model for human rotational perception and the dynamic responses of the SCCs. Additionally, this study provides foundational data to address sensory conflict, spatial disorientation, and various applications that require a precise dynamics model.

## Highlights

• Quantitative analysis of cupula deformation under off-center rotation conditions.
• Identification of differences in semicircular canal dynamic responses between off-center and centered rotations.
• Development of a modified bandpass filter model considering offset distance.
• Providing insights that contribute to understanding sensory conflicts in real and virtual environments.

## 1 Introduction

Spatial orientation, especially rotational perception, is essential for maintaining postural and sensory stability in daily life [1,2]. The semicircular canals (SCCs) within the vestibular system enable the human body to detect rotational motion, including rotational direction, angular velocity, and acceleration [3,4]. Rotational motion can be classified into centered and off-center rotation, depending on the relative position between the axis of rotation and the center of the rotating body [5,6]. Generally, centered rotation refers to a situation where the center of the rotating body coincides with the axis of rotation; otherwise, it is off-center rotation [5]. Previous research has analyzed the dynamics of the SCCs by assuming centered rotation [7,8], and even rotational perception has been mathematically modeled using a bandpass filter under this assumption [9]. However, human vestibular organs are located more than 30 mm away from the body's center [10], and situations where the axis of rotation coincides with the center of the SCC are relatively rare. This indicates that previous studies on SCCs and rotational perception are limited in representing more common rotational situations, and suggests the need for a model that incorporates the offset distance between the rotation axis and the SCCs.

Moreover, off-center rotation occurs in various real-world situations. In most rotational environments, such as driving, flying, sports, and leisure activities, the axis of rotation deviates from the center of the SCC. In this case, the offset distance can significantly affect the response of the vestibular system and cause changes in rotational perception and spatial awareness [11]. Despite the frequent occurrence of off-center rotation in reality and its potential significance, research that considers these conditions remains limited. For instance, a study developed an algorithm using a hexapod manipulator to provide users with more realistic motion sensations but relied on a bandpass filter model that did not account for off-center rotation [12]. The bandpass filter model of the SCCs is based on the torsion pendulum model [9], which describes the cupula displacement in response to angular velocity but does not account for the offset distance [7,8]. Additionally, a recent study analyzed how vestibular sensations and auditory information interact in self-rotational perception but did not account for the distance from the rotation axis [13].

Nevertheless, some attempts have been made to explore the characteristics of off-center rotation. In studies on motion perception and oculomotor responses under off-center rotation, researchers investigated the effects on gravito-inertial cues or oculomotor reflexes by analyzing ocular motor responses [14,15]. However, these studies did not model the off-center dynamics mathematically. Another study, through simulation, found that the strain of the cupula increases as the rotation center moves away from the SCC but analyzed only the strain variation relative to the offset distance [16]. Additionally, a study investigated the sensitivity of the SCCs and otoliths under off-center rotation conditions but focused solely on angular velocity thresholds, noting that these thresholds decrease as the rotational radius increases [17].

The lack of clear modeling approaches in studies of rotational motion perception and dynamic responses of the SCCs is due to the limitations of existing models in explaining a range of rotational conditions. These limitations may further impact applications related to human rotational perception; therefore, a comprehensive analysis of off-center rotational dynamics is fundamental to understanding various related fields. However, both experimental studies and mock-up models are constrained in their ability to accurately describe these dynamic characteristics. Human experiments have ethical and technical limitations that hinder sufficient exploration of SCCs. Observing the internal processes of the SCCs directly is particularly challenging; thus, researchers have utilized indirect methods such as vestibulo-ocular reflex (VOR) measurements or animal experiments to study the responses of the SCCs [[18], [19], [20]]. However, these indirect approaches inherently differ from the actual responses of the SCCs, suggesting that a more accurate and reliable alternative is needed to describe the dynamics of human SCCs. To address these challenges, mock-up models have been developed to replace direct experiments [[21], [22], [23]]. However, the low Young's modulus of the cupula makes it challenging to create a model that accurately represents the SCCs [21,23,24]. Accordingly, there has been an increase in efforts to understand the dynamic characteristics of the SCCs using simulation techniques such as the finite element method (FEM) [16,[25], [26], [27], [28]]. These simulation-based approaches have the advantage of considering the low Young's modulus of the cupula and can effectively account for various rotational conditions. However, few studies have analyzed the transfer function of the SCCs in relation to the offset distance.

Therefore, we performed an analysis using the FEM to investigate the off-center rotational dynamics of SCCs. Additionally, a two-way fluid-structure interaction (FSI) approach was applied to study the interaction between the cupula and endolymph within the SCCs. The rotational conditions were set to three scenarios: step acceleration motion (SAM), step velocity motion (SVM), and sinusoidal motion. SAM was used for comparison with existing literature and model improvement, while SVM and sinusoidal motion were used to confirm the variation in the parameters of the bandpass filter. In the methods section, we detail the formation of the three-dimensional model structure, the simulation parameters, and the process of modeling physical phenomena. Then, twenty-one different rotational conditions are defined. In the results section, we investigate mesh independence and the dynamic response of the SCCs under SAM conditions, and discuss the limitations of existing bandpass filter models based on results obtained with varying offset distances under SVM and sinusoidal motion conditions. Then, we propose a modified transfer function that accounts for the offset distance. Finally, by linking the results to real-world and virtual environments, we provide a comprehensive discussion on the implications of off-center rotation conditions on various application fields related to human rotational perception.

In summary, this study analyzes the effect of off-center rotation on the dynamic characteristics of human SSCs, thereby expanding the understanding of SSC dynamics more broadly and accurately. Our findings contribute to a more generalizable analysis of human rotational perception and the rotational response of the SCCs. Additionally, this study provides foundational data to address sensory conflict, spatial disorientation, and various applications that require a precise dynamics model.

## 2 Methods

### 2.1 Computational model for off-center rotational dynamics

**Fig. 1:**
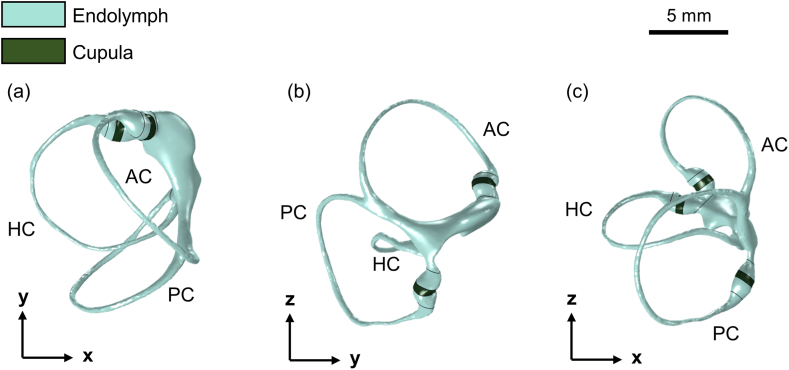
Geometrical model of the left semicircular canals (SCCs) visualized on (a) the xy-plane (top view), (b) the yz-plane (side view), and (c) the xz-plane (back view). The plot depicts three orthogonal SCCs: HC (horizontal canal), AC (anterior canal), and PC (posterior canal). The model is filled with endolymph and includes three distinct cupulae.

**Fig. 2:**
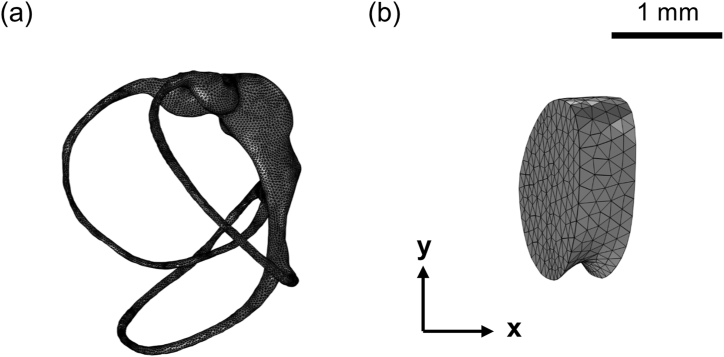
Tetrahedral mesh of the SCCs and HC cupula. (a) Mesh of the entire SCCs, showing the endolymph region with 289,974 elements. (b) Detailed mesh of the HC cupula region, consisting of 5,956 elements.

A reconstructed 3D model (Fig. 1) of the left human SCCs consists of three distinct canals: the horizontal canal (HC), anterior canal (AC), and posterior canal (PC). These canals are shown in the top view (Fig. 1a), with alternative views (Fig. 1b and c) providing additional perspectives. This model was developed using an existing model file specific to *Homo sapiens* [29]. In this model file, only the membranous labyrinth was used, and the duct size was adjusted using Autodesk 3ds Max 2023 software, referencing the commonly used dimensions of the SCCs [30]. This approach allowed for comparison with other simulation studies. Subsequently, the cupula was modeled in the COMSOL Multiphysics 6.1 software. Instead of portraying its complex structure, it was simplified to a cylindrical model with a thickness of 403 μm, consistent with methods in prior research [26,27]. Excluding the cupula region, the model was entirely filled with endolymph. The finalized geometry was then converted to a tetrahedral mesh with element sizes ranging from 40 μm to 120 μm, yielding 289,974 elements for the endolymph region (Fig. 2a) and 21,849 elements for the three cupulae regions (Fig. 2b). The tetrahedral mesh was used to address the intricate curvature of the SCCs and the low Young's modulus of the cupula (5.4 Pa), especially in time-dependent analyses [24]. This mesh type provides high adaptability to complex geometry [31]. It ensures computational stability in FSI simulations by effectively managing substantial deformations in the interface region [32,33]. This strategy promoted convergence while maintaining computational efficiency and facilitated comparisons with other simulation studies employing tetrahedral meshes [[25], [26], [27]].

Material properties are crucial for accurately simulating the cupula and endolymph interactions. Consistent with previous studies [26,27,34,35], we assigned the cupula a density of 1000 kg/m³, Young's modulus of 5.4 Pa, and a Poisson's ratio of 0.3. For the endolymph, we used a density of 1000 kg/m³ and a viscosity of 0.0085 Pa s. Because the low modulus of the cupula leads to significant nonlinear deformation, we started with a time step of 0.001 ms and increased it to a maximum of 1 ms. To maintain the fidelity of the calculated results, a relative tolerance of 0.001 was maintained, and the damped Newton's method was used to assess the interface between the fluid and structural components.

### 2.2 Physics model including two-way FSI model

The membranous labyrinth of the SCCs consists of endolymph and the gelatinous cupula [3,18,36]. When the labyrinth responds to rotation, the endolymph lags behind due to its inertia [36]. This force subsequently deforms the cupula, activating ion channels in the underlying hair cells and sending neural signals to the brain, establishing a link between cupula deformation and rotational perception [9]. To calculate the deformation, we employed the FEM with COMSOL Multiphysics 6.1. Using this software, we developed an FSI model incorporating the Laminar Flow and Solid Mechanics modules to capture the interface deformation between the cupula and endolymph [35]. We also used the Kelvin–Voigt model [37] to analyze the viscoelastic deformation of the cupula. To improve the accuracy of the FSI interface, the arbitrary Lagrangian–Eulerian (ALE) method was used for the fluid domain. Unlike conventional finite element techniques, the ALE method does not anchor the computational system to a fixed space or material, as seen in standard Lagrangian or Eulerian approaches [38]. This flexibility allows the mesh to move autonomously, optimizing the element shape. In contrast, the structural mechanics component relies on a fixed coordinate system that uses derived strains to compute deformed coordinates.

To emulate the natural behavior of the SCCs, the model was adjusted with specific boundary conditions. Within the model, the behavior of the endolymph fluid is governed by the following Navier–Stokes equations:

(1) $$\rho \left(\frac{\partial u}{\partial t}+u\cdot \nabla u\right)-\mu \nabla^{2}u+\nabla p-F=0\text{,}$$

(2) ∇·u=0,

where ***u*** is the fluid velocity vector, *p* is pressure, *ρ* is endolymph density, *μ* is endolymph viscosity, and ***F*** is the fluid volume force. Due to the similarity of endolymph to water [26,27,34], we characterized endolymph as an incompressible, homogeneous, Newtonian viscous fluid. A no-slip condition is also applied at the interface between the endolymph and the membranous labyrinth. At the boundary adjoining the cupula, the no-slip condition was maintained, along with an automatic-from-frame condition. Additionally, a volume force was applied to the endolymph to replicate the rotational behavior of the structure. The volume forces exerted on the entire endolymph domain were:

(3) $$F_{x}=\rho R\frac{\partial^{2}\theta }{\partial t^{2}}\sin\left(\mathrm{atan}\;2\left(y,x\right)\right)\text{,}$$

(4) $$F_{y}=\rho R\frac{\partial^{2}\theta }{\partial t^{2}}\cos\left(\mathrm{atan}\;2\left(y,x\right)\right)\text{,}$$

where *R* is the distance from the rotation axis, and *θ* is the rotation angle; *atan2()* returns a value within the range of −180°–180°, in accordance with the coordinates provided. We set the *z*-axis as the axis of rotation and defined the rotation as ground-parallel; therefore, the current volume forces are not applied in the *z*-direction.

Previous studies on the cupula have demonstrated its gelatinous nature [35,36], so we modeled it as a viscoelastic material. We treated the cupula as nearly incompressible to mitigate the complexities of the highly nonlinear deformation. The strain tensor for the cupula was defined using Lame coefficients, designated as *μ*_*L*_ and *λ*_*L*_, which are related to Young's modulus (*E*) and Poisson's ratio (*ν*) [39]:

(5) $$\mu_{L}=\frac{E}{2\left(1+\nu \right)}\text{,}$$

(6) $$\lambda_{L}=\frac{\nu E}{\left(1+\nu \right)\left(1-2\nu \right)}\text{.}$$

To calculate the interface between the endolymph and the cupula, we applied a two-way FSI model to the current setup. This approach accounts not only for the forces exerted by the endolymph on the cupula but also for the interaction where cupula deformation influences the endolymph flow [16,40]. In particular, when the cupula undergoes large deformations in a short time step or exhibits nonlinear responses, its deformation can significantly impact the fluid flow. Two-way FSI enables the modeling of these nonlinear deformations, making it advantageous for analyses that require the consideration of substantial deformations or complex structural responses [41]. Furthermore, when time-dependent forces are applied, this approach allows for realistic dynamic responses to be obtained. In the two-way FSI model, the coupling conditions between the cupula and endolymph were defined as follows [42]:

(7) $$u_{e}=u_{c}\text{,}$$

(8) $$u_{c}=\frac{\partial d_{c}}{\partial t}\text{,}$$

(9) $$\sigma_{e}\cdot n_{e}=\sigma_{c}\cdot n_{c}\text{,}$$

where *u* is the velocity vector, *d* is the displacement vector, *σ* is the stress tensor, and *n* is the normal vector to the FSI boundary. The subscripts *e* and *c* denote endolymph and cupula, respectively.

### 2.3 Rotational conditions

This study examined the off-center rotational dynamics of the SCCs, with a particular focus on the HC, as humans primarily encounter ground-parallel rotations. This canal has a distinct inclination of approximately 30° upward from the horizontal plane and heightened sensitivity to ground-parallel rotations [26]. Consequently, all rotations examined in this study are ground-parallel.

We defined twenty-one conditions, varying by offset distance (doff) and angular motion type: accelerating, constant, or sinusoidal (see Table 1). In each case, the structure of the SCCs was positioned in the horizontal *xy*-plane as viewed from above, and the direction of rotation was clockwise. The center of the HC is assumed to have an offset of 3 cm from the center of the skull, so in the case where *d*_*off*_ = 3 cm, the rotational axis matches the center of the body; in other cases, the center of the body is different from the rotational axis. Thus, we set the *d*_*off*_ from 3 to 103 cm.

**Table 1:** Twenty-one rotational conditions based on variations in *ω* and *d*_*off*_ and the nature of angular motion.

Condition	Angular motion	*Δx* (cm)	*dω/dt* or *ω*	Code
1	SAM	3	*dω/dt* = 10°/s^2^	A3·10
2	SAM	3	*dω/dt* = 20°/s^2^	A3·20
3	SAM	3	*dω/dt* = 30°/s^2^	A3·30
4	SVM	3	*ω* = 10°/s	V3·10
5	SVM	3	*ω* = 20°/s	V3·20
6	SVM	3	*ω* = 30°/s	V3·30
7	SVM	13	*ω* = 10°/s	V13·10
8	SVM	23	*ω* = 10°/s	V23·10
9	SVM	33	*ω* = 10°/s	V33·10
10	SVM	43	*ω* = 10°/s	V43·10
11	SVM	53	*ω* = 10°/s	V53·10
12	SVM	103	*ω* = 10°/s	V103·10
13	Sinusoidal	3	*ω*_*P-P*_ = 1°/s	S3·1
14	Sinusoidal	3	*ω*_*P-P*_ = 2°/s	S3·2
15	Sinusoidal	3	*ω*_*P-P*_ = 3°/s	S3·3
16	Sinusoidal	13	*ω*_*P-P*_ = 1°/s	S13·1
17	Sinusoidal	23	*ω*_*P-P*_ = 1°/s	S23·1
18	Sinusoidal	33	*ω*_*P-P*_ = 1°/s	S33·1
19	Sinusoidal	43	*ω*_*P-P*_ = 1°/s	S43·1
20	Sinusoidal	53	*ω*_*P-P*_ = 1°/s	S53·1
21	Sinusoidal	103	*ω*_*P-P*_ = 1°/s	S103·1

Conditions are coded as [A, V, or S] *d*_*off*_ · [acceleration, velocity, or peak-to-peak velocity] (last column, Table 1). Conditions 1 (Code A3·10), 2 (A3·20), and 3 (A3·30) were used to study the dynamic response associated with different accelerating motions (SAM). Conditions 4 (V3·10), 5 (V3·20), and 6 (V3·30) considered the effect of varied angular velocities (SVM) at constant *d*_*off*_ = 3 cm. Conditions 7 (V13·10) to 12 (V103·10) were used to evaluate the time constant of the SCC at a constant angular velocity, but different *d*_*off*_. Conditions 13 (S3·1), 14 (S3·2), and 15 (S3·3) considered the effect of varied peak-to-peak angular velocities (Sinusoidal motion) at constant *d*_*off*_ = 3 cm. Conditions 16 (S13·1) to 21 (S103·1) were used to evaluate the gain factor of the SCC at varied peak-to-peak angular velocities with different *d*_*off*_ values.

### 2.4 Bandpass filter model of rotational motion

In this study, we employed a mathematical model to represent the dynamic behavior of the SCC in response to rotational motion. Specifically, we utilized a bandpass filter model to describe the relationship between the cupula deflection, *δ*, and the head angular velocity, *ω*. This model, originally proposed by Steinhausen [7] and further refined by Van Egmond et al. [8], is widely recognized for capturing the core characteristics of SCC responses to rotational stimuli. The model is expressed as follows [9]:

(10) $$\frac{\delta }{\omega }=\frac{Ks}{\left(\tau_{L}s+1\right)\left(\tau_{S}s+1\right)}$$

where *K* is the gain, *s* represents the Laplace operator, *τ*_*L*_ is the long time constant, and *τ*_*S*_ is the short time constant.

However, this traditional model assumes a fixed rotation axis centered within the SCC, allowing cupula deflection to be measured as an angular displacement around this axis [8]. In scenarios where the rotation axis shifts or moves, the nature of cupula movement changes; thus, a different measurement approach is needed. Instead of angular displacement, actual deformation (i.e., displacement) provides a more accurate representation of cupula deflection. Since the existing *δ* only captures one-dimensional angular displacement, this study employs vertical displacement from the cupula's cross-section as a more comprehensive measure of deflection, similar to the approach used in a previous study [43].

The time constants, *τ*_*L*_ and *τ*_*S*_, were determined based on indirect experimental observations and subjective sensation measurements for human participants under controlled rotational stimuli [18]. Specifically, the *τ*_*S*_ is a parameter that addresses the rapid adaptation of the cupula; however, using the value in previous studies, this value was fixed at 0.01, as this study focuses on common rotational motions encountered in daily life rather than high-frequency responses [9]. In contrast, the long time constant is used to describe the sustained interaction between the cupula and the endolymph and can be measured as the time constant for the cupula's response to SVM. This study considers the gain factor at a specific operating point rather than gain across the entire frequency range. This approach is intended to compare the changes in the gain factor at this operating point to assess the dynamic characteristics induced by off-center rotation. Accordingly, the operating frequency was set to 10 Hz, within the passband of the filter, and the ratio of the output to the sinusoidal input was measured [44].

## 3 Results

### 3.1 Cupula deformation and mesh independence analysis in SAM conditions

**Fig. 3:**
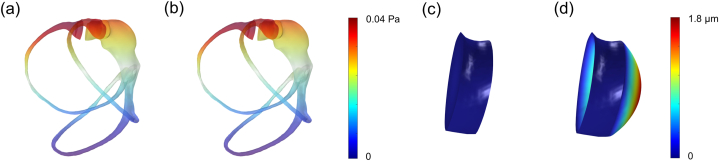
Temporal analysis of endolymph pressure distribution and HC cupula deformation under A3·10 condition. (a) Endolymph pressure distribution at 0.02 s, and (b) at 8 s, with a shared color legend. (c) HC cupula deformation at 0.02 s, and (d) at 8 s, visualized with a shared color map. For clear visualization, cupula deformation is scaled up by a factor of 200.

The first part of the results focuses on examining the deformation patterns of the cupula under SAM conditions and verifying the reliability of the simulation results through mesh independence analyses. SAM applies a continuous rotational force to the SCCs for 10 s, causing the endolymph within the canal to experience force in a consistent direction. Across all step acceleration conditions, the fluid pressure distribution at the initial and subsequent time points remained consistent (Fig. 3a and b). This consistency indicates that the force exerted on both sides of the cupula remains constant over time. It also suggests that the pressure distribution and sustained force are maintained regardless of time, allowing the SCCs to reach an equilibrium state under SAM conditions.

When rotation begins, the membranous labyrinth moves with the person's motion, while the endolymph resists movement due to inertial forces. Therefore, initial cupula deformation under SAM conditions is induced by the inertia of the endolymph. Over time, the endolymph moves in tandem with the labyrinth's movement, and the equilibrium state of endolymph pressure leads to a differential pressure distribution on both sides of the cupula, resulting in a directional deformation. SAM finally establishes an equilibrium between the flow of the endolymph and the interaction between the endolymph and the cupula. The interaction between these two domains is modeled as a two-way FSI with automatic-from-frame boundary conditions. Consequently, deformation of the HC cupula does not occur at the initial time point (0.02 s, Fig. 3c) but becomes apparent at a later time point (8 s, Fig. 3d). This deformation reaches a saturation point when the fluid force applied to the cupula reaches equilibrium, and subsequently, the deformation stabilizes at its maximum value. These findings align with previous studies [26,28].

**Fig. 4:**
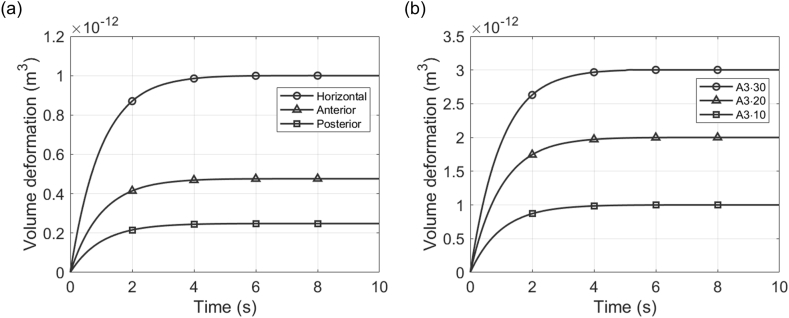
Temporal analysis of volume deformation in the cupula under step acceleration motion (SAM) conditions. (a) Volume deformation over time for HC, AC, and PC under the A3·10 condition, showing that HC reaches equilibrium with the largest deformation, followed by AC and PC. (b) Volume deformation over time under three rotational conditions (A3·10, A3·20, A3·30), demonstrating an increase in maximum deformation with higher acceleration.

**Fig. 5:**
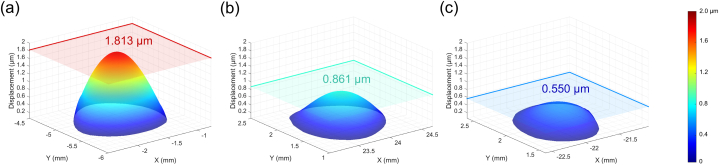
Three-dimensional surface plots of cupula volume deformation at 8 s under the A3·10 condition, illustrating maximum displacement for each canal. Each plot indicates the maximum displacement point and corresponding plane for (a) HC, (b) AC, and (c) PC, with a shared color map. These plots provide a detailed visualization of unidirectional volume changes and maximum displacement values.

In the A3·10 condition presented in the previous section, the deformation over time for HC, AC, and PC shows that HC reaches equilibrium with the largest deformation values, followed by AC and PC (Fig. 4a). Using the unidirectional deformation characteristic of the cupula, the volume deformation of the cupula is measured based on the protruding surface. The maximum deformation values in each canal are measured at 1.00 × 10^−12^, 0.48 × 10^−12^, and 0.25 × 10^−12^ m^3^, respectively. All three canals exhibit no initial deformation, followed by a rapid increase that gradually slows until an equilibrium state is reached. All rotation conditions in this study use the z-axis as the rotation axis because humans primarily experience horizontal rotations in real-life scenarios; thus, all rotation conditions represent ground-parallel rotation. Consequently, HC deformation is the most significant, followed by the other two SCCs. Additionally, when these results are depicted as three-dimensional surface plots, the unidirectional volume changes of the cupula can be observed in more detail (Fig. 5). These plots display the volume deformation and maximum cupula displacement for HC (Fig. 5a), AC (Fig. 5b), and PC (Fig. 5c). Thus, not only the volume changes but also the largest displacement of the cupula are identified. The maximum displacement values in each canal are measured at 1.813, 0.861, and 0.550 μm, respectively.

Deformation of the HC cupula under different SAM conditions was also calculated (Fig. 4b). In particular, the maximum deformation values at equilibrium for the A3·10, A3·20, and A3·30 conditions were measured as 1.00 × 10^−12^, 2.01 × 10^−12^, and 3.02 × 10^−12^ m^3^, respectively. This proportionality between maximum deformation values and acceleration demonstrates that the HC's response increases proportionally with the acceleration of ground-parallel rotations, despite the interconnectedness of the endolymph across all SCCs; this finding aligns well with existing simulation research [26,28].

**Fig. 6:**
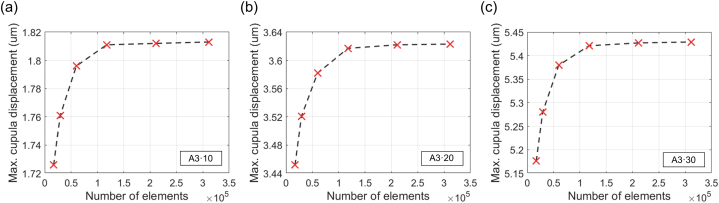
Analysis of mesh independence based on the maximum displacement of the HC cupula at 8 s under three SAM rotational conditions. Each plot shows the relationship between the number of mesh elements and maximum cupula displacement for (a) A3·10, (b) A3·20, and (c) A3·30 conditions. Red cross marks indicate data points corresponding to six different mesh conditions. Displacement values stabilize at approximately 100,000 elements, confirming mesh independence.

Based on these findings, mesh independence was analyzed using the maximum displacement of the HC cupula under three SAM rotational conditions (Fig. 6). A total of six mesh conditions were employed, with specific mesh information detailed in Table 2. For all three conditions (A3·10, Fig. 6a; A3·20, Fig. 6b; A3·30, Fig. 6c), the displacement values stabilized when the mesh number was approximately 100,000 or larger, confirming mesh independence. This ensures the convergence and consistency of the simulations and, thus, reinforces the reliability of the model.

**Table 2:** Mesh specifications used for analyzing mesh independence under three SAM rotational conditions.

Mesh Cond.	Max. element size (μm)	Min. element size (μm)	Number of elements	Max. Disp. in A3·10 (μm)	Max. Disp. in A3·20 (μm)	Max. Disp. in A3·10 (μm)
1	360	240	16,982	1.726	3.452	5.176
2	280	180	29,256	1.761	3.521	5.280
3	240	120	59,837	1.796	3.582	5.380
4	200	80	117,535	1.811	3.617	5.421
5	150	50	211,245	1.812	3.622	5.427
6	120	40	311,823	1.813	3.623	5.429

### 3.2 Cupula deformation under fixed and varying offset distances in SVM conditions

**Fig. 7:**
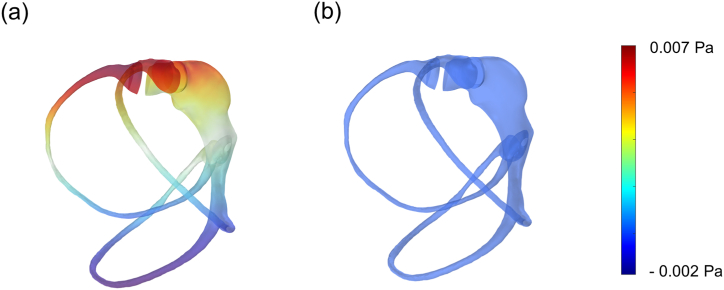
Temporal analysis of endolymph pressure distribution under V3·10 condition. (a) Endolymph pressure distribution at 0.02 s, and (b) at 8 s, with a shared color legend. Over time, the rotational forces dissipate, resulting in no significant pressure differences within the endolymph.

**Fig. 8:**
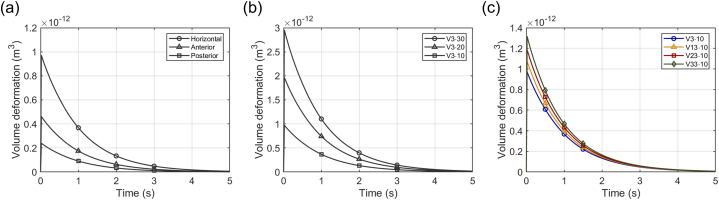
Temporal analysis of volume deformation in the cupula under step velocity motion (SVM) conditions. (a) Volume deformation over time for HC, AC, and PC under the V3·10 condition, with HC showing the highest peak deformation, followed by AC and PC. (b) Volume deformation over time with a fixed offset distance (*d*_*off*_) under three rotational conditions (V3·10, V3·20, V3·30), illustrating that higher *ω* leads to increased peak deformation. (c) Volume deformation over time with varying *d*_*off*_ values under four rotational conditions (V3·10, V13·10, V23·10, V33·10), showing that peak deformation increases as *d*_*off*_ increases.

Unlike SAM conditions, the SCCs begin to rotate instantaneously under SVM conditions with an angular velocity *ω*. Consequently, this condition generates the maximum torque at the onset of rotation. Initially, the pressure distribution within the endolymph is distinct and tends to decrease gradually over time. For instance, under the V3·10 condition, the pressure distribution observed at the initial time nearly disappears by the final time (Fig. 7a and b). This indicates that the torque diminishes over time, resulting in no significant pressure difference within the endolymph. The volume deformation results of each SCC under this condition showed peak deformation caused by the initial torque (Fig. 8a). Specifically, the HC exhibited the highest peak deformation, followed by the AC and PC. This pattern is similar to SAM conditions because the rotation is ground-parallel. The peak deformation values were measured as 0.97 × 10^−12^, 0.46 × 10^−12^, and 0.24 × 10^−12^ m^3^, respectively, and each canal's deformation gradually decreased after reaching the peak.

In this study, the SVM rotation conditions with fixed offset distances were V3·10, V3·20, and V3·30. The volume deformation results of the HC cupula in each case showed that peak deformation increased with increasing angular velocity *ω*, with the most pronounced deformation observed under the V3·30 condition (Fig. 8b). The peak deformation values were 0.97 × 10^−12^, 1.95 × 10^−12^, and 2.95 × 10^−12^ m^3^, and the peak displacement values were 1.763, 3.541, and 5.350 μm, respectively. The instantaneous torque under SVM conditions induces a rapid inertial response in the endolymph, causing significant initial deformation of the cupula. This indicates that sudden rotation from a stationary state amplifies the interaction between the endolymph and the cupula, leading to substantial changes in the pressure distribution. Under SAM conditions, *ω* increases gradually, resulting in gradual deformation. In contrast, deformation occurs immediately under SVM conditions due to the rapid initial increase in *ω*. Consequently, SAM conditions achieve stable deformation over time, whereas SVM conditions exhibit a pattern of rapidly decreasing deformation after the initial peak. These findings illustrate how the differences in rotational characteristics between the two conditions affect the interaction between the cupula and the endolymph. In this case, the time constant *τ*_*L*_ is determined by measuring the time taken to reach 37 % of the peak displacement (Table 3). The *τ*_*L*_ for V3·10, V3·20, and V3·30 were calculated as 1.025, 1.028, and 1.030 s, respectively. The calculated time constants showed similar values with minimal variation.

**Table 3:** Peak deformation, peak displacement, 37 % peak displacement, and *τ*_*L*_ values for fixed *d*_*off*_ conditions.

Code	Peak deformation (m^3^)	Peak displacement (μm)	37 % peak displacement (μm)	*τ*_*L*_ (s)
V3·10	0.97 × 10^−12^	1.763	0.652	1.025
V3·20	1.95 × 10^−12^	3.541	1.310	1.028
V3·30	2.95 × 10^−12^	5.350	1.980	1.030

Results for four different *d*_*off*_ values, rather than a fixed *d*_*off*_, were analyzed similarly. The four conditions, including V3·10, involved rotations with identical *ω*. The volume deformation results of the HC cupula in each case showed that peak deformation increased with increasing offset distance, with the most pronounced deformation observed under the V33·10 condition (Fig. 8c). The peak deformation values were 0.97 × 10^−12^, 1.07 × 10^−12^, 1.18 × 10^−12^, and 1.32 × 10^−12^ m³, respectively. This suggests that when the same step velocity is applied, increasing distances from the rotation axis lead to larger volume deformations. Additionally, *τ*_*L*_ was calculated for these conditions (Table 4), allowing for a comparison of the influence of *d*_*off*_. The *τ*_*L*_ for V3·10, V13·10, V23·10, and V33·30 were calculated as 1.025, 1.004, 0.984, and 0.959 s, respectively. These results, unlike those with the same *d*_*off*_, show significant differences in *τ*_*L*_ across conditions, with the time constant decreasing as the *d*_*off*_ increases. For identical distances, the time constant showed minimal variation despite changes in rotation conditions. However, increasing the distance from the rotation axis resulted in changes to *τ*_*L*_ even when *ω* remained the same. Therefore, the existing bandpass model, which assumes a constant distance from the rotation axis, can analyze the centered rotational dynamics of SCCs using a single time constant. However, for systems where the distance from the rotation axis varies, the existing model may not accurately reflect these dynamic characteristics.

**Table 4:** Peak deformation, peak displacement, 37 % peak displacement, and *τ*_*L*_ values for varying *d*_*off*_ conditions.

Code	Peak deformation (m^3^)	Peak displacement (μm)	37 % peak displacement (μm)	*τ*_*L*_ (s)
V3·10	0.97 × 10^−12^	1.763	0.652	1.025
V13·10	1.07 × 10^−12^	1.973	0.730	1.004
V23·10	1.18 × 10^−12^	2.223	0.823	0.984
V33·10	1.32 × 10^−12^	2.514	0.930	0.959

### 3.3 Cupula deformation under fixed and varying offset distances in sinusoidal conditions

This section examines the results under sinusoidal motion conditions, with a particular focus on the gain factor of the bandpass filter. The study sets the operating frequency at 10 Hz within the passband to analyze changes in the gain factor at this operating point. The gain factor is defined as the ratio of the displacement amplitude *δ*_*amp*_ to the amplitude of the angular velocity *ω*_*amp*_ of the sinusoidal input [44].

**Fig. 9:**
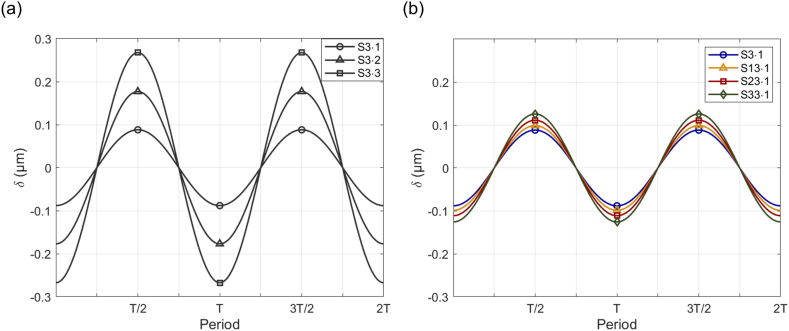
Sinosoidal displacement (*δ*) over two cycles (2T) under sinusoidal rotation conditions. (a) Displacement amplitude (*δ*_*amp*_) of the HC cupula for fixed *d*_*off*_ conditions (S3·1, S3·2, S3·3), showing increased *δ*_*amp*_ with rising angular velocity amplitude (*ω*_*amp*_). (b) *δ*_*amp*_ under varying *d*_*off*_ conditions (S3·1, S13·1, S23·1, and S33·1), illustrating that *δ*_*amp*_ increases as *d*_*off*_ increases, indicating a more pronounced cupula deformation as the SCC structure moves further from the axis of rotation.

The outcomes of sinusoidal rotation under conditions with fixed *d*_*off*_ (S3·1, S3·2, S3·3) indicate that the *δ*_*amp*_ of the HC cupula increases as the *ω*_*amp*_ rises (Fig. 9a). The gain factors, calculated based on the amplitude of each input and displacement, are 0.1764, 0.1771, and 0.1783 μm (°/s)^−1^, respectively, as summarized in Table 5. These results suggest that the gain factor remains relatively stable in systems operating at the same axis and frequency. This consistency indicates that these differing conditions can be analyzed using a single bandpass model, as they maintain a stable output ratio relative to the input angular motion.

**Table 5:** *ω*_*amp*_, *δ*_*amp*_, and gain factor for fixed *d*_*off*_ conditions.

Code	*ω*_*amp*_ (°/s)	*δ*_*amp*_ (μm)	Gain factor (μm (°/s)^−1^)
S3·1	0.5	0.0882	0.1764
S3·2	1.0	0.1771	0.1771
S3·3	1.5	0.2675	0.1783

The results under varying *d*_*off*_ conditions were analyzed similarly (Fig. 9b). Consistent with previous analyses, four conditions (S3·1, S13·1, S23·1, and S33·1) were compared. In each scenario, a sinusoidal rotational input with identical frequency and *ω*_*amp*_ was applied. The deformation results revealed that, despite the uniform rotational input, the *δ*_*amp*_ differed across the conditions. Specifically, the measured *δ*_*amp*_ values were 0.0882, 0.0987, 0.1112, and 0.1256 μm, respectively. Consequently, the calculated gain factors were determined to be 0.1764, 0.1974, 0.2224, and 0.2512 μm (°/s)^−1^, as shown in Table 6. These findings indicate that as *d*_*off*_ increases, meaning the SCC structure moves further from the axis of rotation, the deformation of the cupula becomes more pronounced. This suggests that even with identical rotational input, the output can vary significantly, implying that the existing bandpass filter model alone is insufficient to fully explain the cupula deflection resulting from rotation.

**Table 6:** *ω*_*amp*_, *δ*_*amp*_, and gain factor for varying *d*_*off*_ conditions.

Code	*ω*_*amp*_ (°/s)	*δ*_*amp*_ (μm)	Gain factor (μm (°/s)^−1^)
S3·1	0.5	0.0882	0.1764
S13·1	0.5	0.0987	0.1974
S23·1	0.5	0.1112	0.2224
S33·1	0.5	0.1256	0.2512

### 3.4 Comparison of bandpass filter parameters for centered vs. off-center rotations

In the preceding section, we established that the time constant (τ) and gain factor of the conventional bandpass filter effectively represent various rotational dynamic characteristics without significant variation when the offset distance (*d*_*off*_) is fixed. This finding indicates that the system's dynamic behavior remains stable when the rotation axis is fixed. However, these parameters are significantly affected when the distance from the rotation axis changes. Specifically, as *d*_*off*_ increases, *τ*_*L*_ decreases, and the gain factor at the operating point tends to rise.

**Fig. 10:**
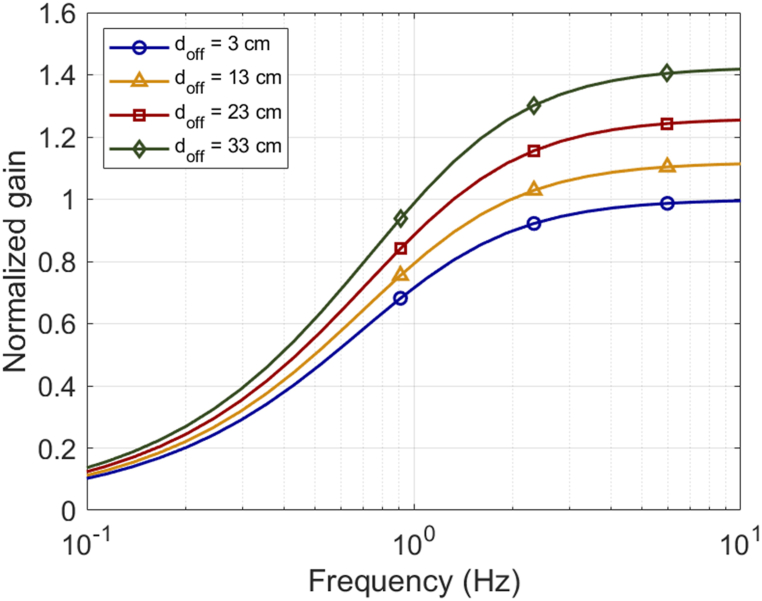
Normalized frequency response of the gain, with *d*_*off*_ = 3 cm set as the reference. This plot illustrates the variations in gain across different *d*_*off*_ values, highlighting how the varying *d*_*off*_ affects the dynamic responses of the SCCs.

**Fig. 11:**
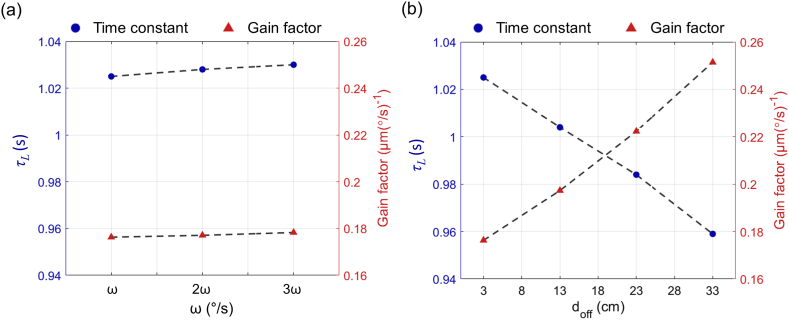
Influence of *d*_*off*_ on *τ*_*L*_ and gain factor in the bandpass filter model for SCCs. (a) *τ*_*L*_ and gain factors plotted for scenarios with fixed *d*_*off*_, according to angular velocity magnitude ratios. (b) Comparative analysis of *τ*_*L*_ and gain factor across varying *d*_*off*_ conditions. Both plots clearly demonstrate the impact of *d*_*off*_ on the dynamic parameters, suggesting that a single time constant and gain factor are insufficient to describe the SCCs' dynamics under varying *d*_*off*_.

To clarify the changes in the dynamic characteristics of the SCC as *d*_*off*_ increases, we plotted the normalized gain in the frequency response based on the previous data (Fig. 10). This graph was normalized with *d*_*off*_ = 3 cm as a reference, allowing for comparison with other *d*_*off*_ conditions. The results indicate that the variations in the time constant and gain factor due to changes in the distance from the rotation axis are not sufficient to describe the rotational dynamic characteristics of the SCCs using a single bandpass filter model. Furthermore, to analyze in detail how the time constant and gain factor change with the distance from the rotation axis, a comparative analysis was conducted under two scenarios. First, under conditions with no change in *d*_*off*_, the *τ*_*L*_ corresponding to each velocity magnitude ratio (V3·10, V3·20, V3·30) and the gain factors for the S3·1, S3·2, and S3·3 conditions were plotted (Fig. 11a). Second, under conditions with varying *d*_*off*_ from the rotation axis, the *τ*_*L*_ for the V3·10, V13·10, V23·10, and V33·10 conditions and gain factors for the S3·1, S13·1, S23·1, and S33·1 conditions were plotted according to *d*_*off*_ (Fig. 11b). These two results display *τ*_*L*_ and gain factor on the left and right axes, respectively, with identical ranges to facilitate comparison. This arrangement clearly demonstrates the influence of *d*_*off*_ on the system's dynamic characteristics. Consequently, the analysis confirms that *d*_*off*_ substantially affects the primary parameters of the band-pass filter model. This finding implies that incorporating the distance from the rotation axis is essential for accurately assessing the dynamic characteristics of the SCCs in system modeling.

### 3.5 Modified bandpass filter model

Through the preceding results, we have established the necessity of a bandpass filter model that accounts for the influence of *d*_*off*_. Consequently, in this section, we develop a model that represents the time constant *τ*_*L*_ and gain factor based on varying *d*_*off*_, and integrate it into the existing bandpass filter model. To accurately fit each parameter, additional data points were utilized, specifically for *d*_*off*_ values of 43 cm, 53 cm, and 103 cm. Applying the same analytical methods as before, we calculated the gain factor and *τ*_*L*_ for these distances, with the results summarized in Table 7. Incorporating these values allowed us to model the changes in bandpass parameters in more detail as a function of *d*_*off*_. We applied linear, polynomial, and exponential approximations to model both parameters and compared the results using mean squared error (MSE). The most suitable model was then incorporated into the bandpass model.

**Table 7:** Additional results for optimal approximation across various *d*_*off*_ values.

*d*_*off*_ (cm)	*ω*_*amplitude*_ (°/s)	*δ*_*amplitude*_ (μm)	Gain factor (μm (°/s)^−1^)	*τ*_*L*_ (s)
43	0.5	0.1402	0.2804	0.940
53	0.5	0.1565	0.3130	0.921
103	0.5	0.2492	0.4984	0.803

**Fig. 12:**
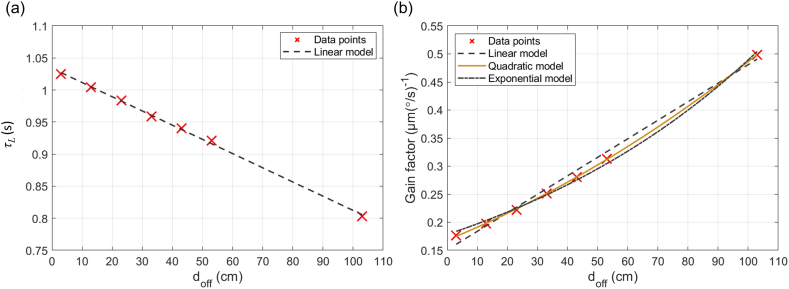
(a) Linear approximation of *τ*_*L*_ as a function of *d*_*off*_, displaying minimal mean squared error (MSE) and ensuring computational simplicity. (b) Comparison of three approximations of the gain factor relative to *d*_*off*_, showing a significantly lower MSE in the polynomial model compared to linear and exponential models, enabling a more accurate representation of gain changes with increasing *d*_*off*_.

The results for *τ*_*L*_ were adequately represented by a linear model (Fig. 12a). Specifically, the MSE values for the linear, polynomial, and exponential models were 5.73 × 10^−6^, 2.34 × 10^−6^, and 4.95 × 10^−6^ s^2^, respectively. Although the polynomial model exhibited the lowest MSE, indicating a better fit, it introduces unnecessary complexity by adding a quadratic term. Given that all three models demonstrated very small errors, opting for the simpler linear model is preferable to avoid unnecessary complexity. In contrast, the modeling of the gain factor revealed different trends (Fig. 12b). Similar to the time constant, we applied linear, polynomial, and exponential approximations. The MSE values for these models were 9.86 × 10^−5^, 9.18 × 10^−7^, and 3.36 × 10^−5^ (μm (°/s)^−1^)^2^, respectively. These results indicate that the polynomial model is significantly more suitable compared to the linear and exponential approaches. While a linear model might be chosen to reduce complexity depending on the *d*_*off*_ being considered, the polynomial model is a better choice when precise modeling of distance-dependent variations is required. Based on these findings, we designed a modified bandpass filter model that incorporates the influence of *d*_*off*_, the distance from the rotation axis to the SCCs. The proposed model is presented as follows:

(11) $$\frac{\delta }{\omega }=\frac{\left(1+\beta_{1}d+\beta_{2}d^{2}\right)Ks}{\left(\left(1-\alpha d\right)\tau_{L}s+1\right)\left(\tau_{S}s+1\right)}$$

where *K* is the gain for centered rotation, *τ*_*L*_ is the long time constant for centered rotation, *τ*_*S*_ is the short time constant, *d* is the offset distance, and *s* is the Laplace operator. The constants in this model are *α* = 0.002143 cm^−1^, *β*_*1*_ = 0.01295 cm^−1^, and *β*_*2*_ = 0.000059 cm^−2^. This allows for the application of the modified bandpass filter model under various conditions by adjusting for different *d*_*off*_ values.

The proposed modified model addresses the limitations of conventional bandpass filter models that assume a fixed rotation axis, enabling more accurate predictions in systems where the distance between the rotation axis and the SCCs varies significantly. Specifically, this model accounts for the dynamic characteristics that arise under off-center rotation conditions, making it highly applicable to various future applications and simulation studies related to the SCCs. However, the proposed model has certain limitations. Since these findings are based on simulation results, it is essential to understand the trends in dynamic characteristics associated with varying *d*_*off*_ and incorporate these into further research. To apply this model to actual systems, more realistic modeling and an integrated approach will be necessary. Nonetheless, this study holds significant value as an initial effort to quantitatively analyze the impact of *d*_*off*_ on the rotational dynamic characteristics of the SCCs. It is expected that the proposed model will serve as an important reference for future research.

## 4 Discussion

The findings of this study highlight the importance of a modified bandpass filter model that considers the influence of the distance between the rotation axis and the SCCs. Unlike conventional models that assume a fixed rotation axis centered within the SCCs, our modified model incorporates varying *d*_*off*_, thereby improving the model's ability to represent systems where the rotation axis deviates from the center of the SCC. This improvement is especially relevant since most real-life rotational scenarios involve off-center rotations, which were not fully represented in previous studies [[7], [8], [9],[12], [13], [14], [15], [16], [17]].

Our results demonstrate that as *d*_*off*_ increases, the *τ*_*L*_ decreases, and the gain factor at the operating point increases. This trend indicates that the dynamic response of the SCCs varies with offset distances, revealing the limitations of the traditional bandpass filter model under these conditions. Fig. 10a illustrates the normalized gain variations, showing that the conventional model does not represent the changes in SCC dynamics caused by varying *d*_*off*_. Additionally, Figs. 10b and c provide a comparative analysis of *τ*_*L*_ and gain factors under fixed and varying *d*_*off*_ conditions, respectively. These analyses confirm that a single time constant and gain factor are insufficient to describe the rotational dynamics when *d*_*off*_ is not constant, thereby validating the necessity of a modified model.

The modified model developed in this study has significant potential and implications for more accurately explaining and predicting situations where sensory conflict intensifies or lessens under specific conditions that were previously unresolved. This model has valuable implications across various fields, helping address sensory conflict [45], spatial disorientation [46], and motion perception issues. For example, in autonomous vehicles, passengers often experience motion sickness due to vestibular stimulation without corresponding visual cues [47,48]. Similarly, astronauts in microgravity environments and pilots in aviation frequently face spatial disorientation and motion sickness from off-center rotational dynamics that traditional models cannot adequately capture [49,50]. By incorporating offset distance, our model allows for a more precise analysis of SCC dynamics under such conditions, offering valuable insights for designing vehicle motion algorithms, spacecraft protocols, and aviation training methods that can mitigate sensory conflicts. In virtual environments, such as virtual reality (VR), users frequently experience cybersickness due to mismatches between visual motion cues and their vestibular system's expectations [51]. Our model guides the development of VR systems that better align with human vestibular responses, thus reducing cybersickness and enhancing user experience. Lastly, in sports science, where athletes undergo complex, multi-axis rotations, understanding how off-center rotations impact balance and spatial orientation can inform training programs that improve performance and reduce injury risk [52]. Altogether, accounting for offset distance plays a crucial role in effectively predicting and addressing sensory conflicts in both real-world and virtual environments.

This study analyzed the dynamic characteristics of the SCCs through simulations; however, it lacks experimental validation. As mentioned in the introduction, directly observing the internal processes of human SCCs is highly challenging due to ethical and technical constraints. In addition, mock-up models still have limitations. Therefore, this study used the FEM and a two-way FSI approach to model the dynamics of the SCCs. While these simulation-based methods offer valuable insights, they may not fully capture the complexities inherent in biological systems. Therefore, rather than aiming to fully validate and apply the model as is, it is important to consider the effects of offset distance based on these findings. To address the current limitations and enhance the reliability of the model, future research will focus on experiment-based validation. Specifically, leveraging the simulation results from this study, we plan to conduct research aimed at accurately and precisely assessing and quantitatively solving sensory conflicts, particularly those occurring in VR environments. This will contribute to improving VR system design and enhancing user experience by reducing cybersickness. Additionally, we intend to integrate non-linear dynamics and machine learning algorithms to further enhance the model's predictive capabilities and to understand the interactions between *d*_*off*_ and individual vestibular sensitivities more precisely. This will provide essential data for the development of personalized applications. Furthermore, expanding the model to include three-dimensional rotational dynamics and multi-axis rotation conditions will enable a more comprehensive and in-depth understanding of SCC responses. Collaborations with interdisciplinary fields such as biomechanics, neuroscience, and computer science will facilitate the continuous improvement of the model and its applicability across diverse real-world and virtual scenarios. This research will serve as a vital tool for accurately understanding and addressing the dynamic responses of the SCCs in various applications.

## 5 Conclusion

In summary, this study highlights the importance of considering the distances between the rotation axis and the SCCs in modeling the dynamic responses of the human SCCs. By developing a modified bandpass filter model that accounts for varying the distance, we have provided a more accurate model for representing SCC behavior under off-center rotational conditions. Despite certain limitations, our findings offer valuable insights that can enhance applications ranging from autonomous driving and aerospace to VR and sports training. This research provides a foundation for further studies to refine vestibular models and enhance our understanding of human rotational perception and spatial orientation.

## CRediT authorship contribution statement

**Sion Cha:** Writing – original draft, Visualization, Methodology, Investigation, Formal analysis, Data curation, Conceptualization. **Wooksung Kim:** Writing – review & editing, Supervision, Investigation, Conceptualization.

## Data availability statement

Data will be made available on request.

## Declaration of competing interest

The authors declare that they have no known competing financial interests or personal relationships that could have appeared to influence the work reported in this paper.
